# Posterior Positivity Distribution Analysis of Subclinical Bluetongue in the Eastern and North-Eastern States of India: A Wakeup Call for Outbreak Preparedness

**DOI:** 10.3390/v17010018

**Published:** 2024-12-26

**Authors:** Siddhartha Narayan Joardar, Aritra Sanyal, Ahmed Abd El Wahed, Saibal Ray

**Affiliations:** 1 Department of Veterinary Microbiology, Faculty of Veterinary and Animal Sciences, West Bengal University of Animal and Fishery Sciences, 37 Kshudiram Bose Sarani, P.O. Belgachia, Kolkata 700 037, West Bengal, India; joardar69@gmail.com; 2 Institute of Astronomy, Space and Earth Science (IASES), P 177, CIT Road, Kolkata 700 054, West Bengal, India; 3 Institute of Animal Hygiene and Veterinary Public Health, University of Leipzig, An den Tierkliniken, D-04103 Leipzig, Germany; 4 Centre for Cosmology, Astrophysics and Space Science (CCASS), GLA University, Mathura 281 406, Utter Pradesh, India; saibal.ray@gla.ac.in

**Keywords:** bluetongue, ELISA, north-eastern states, sero-epidemiology, cattle, goat, sheep

## Abstract

Bluetongue (BT) is considered endemic in the southern states of India, with sporadic incidences reported from the northern, western and central parts of India. However, the eastern and north-eastern states of India have not experienced active disease so far. In the recent past, an extensive sero-epidemiological investigation was carried out in the eastern and north-eastern Indian states. With the aim of getting updated and refined estimates of positivity rates, the sero-surveillance data were analyzed using the Markov chain Monte Carlo (MCMC) method to calculate the positivity rates of various species across different states and agro-climatic zones. The posterior positivity distribution helped in accurately estimating the seroprevalence of bluetongue virus (BTV) among different species and regions. The MCMC method was applied for the first time in a BTV seroprevalence analysis that enhanced our understanding of infection dynamics, guided targeted interventions and supported better decision-making in bluetongue disease control, prevention and disease preparedness. This exercise is quite pertinent in the context of the recent upsurge of newer BTV strains, e.g., BTV-3 and BTV-8, in the western world. In short, as a powerful computational tool, MCMC could be used for accurate seroprevalence estimation, species-specific insights, regional analysis, enhanced decision-making and epidemiological insights for bluetongue.

## 1. Introduction

Bluetongue (BT), an arthropod (*Culicoides* midges) vector-borne viral disease, affects domestic as well as wild ruminants. It is commonly seen in sheep, goats and cattle. However, BT infects other domestic and wild animals, viz. water buffaloes, camels, elephants, elk, white tailed deer and blesbok [1]. The first outbreak of BT occurred in Cyprus in 1924 [2]. Maharashtra experienced the first BT incidence in India in the year 1964. The report was based on the detection of anti-bluetongue antibodies in small ruminants as well as in combination with their clinical signs [3]. Different states of northern India(Jammu and Kashmir, Himachal Pradesh, Punjab, Haryana, Uttar Pradesh and Rajasthan), central India(Madhya Pradesh), western India(Maharashtra and Gujarat) and southern India(Karnataka, Andhra Pradesh and Tamil Nadu)have reported outbreaks of BT. However, no BT incidences or outbreaks have been officially declared in eastern or north-eastern India [4,5,6,7,8].

Bluetongue virus (BTV) (Family: *Reoviridae*, Genus: *Orbivirus*), the agent of bluetongue disease (BT) in ruminants, is transmitted by blood-feeding *Culicoides* spp. (Diptera) [9]. *Culicoides* midges remain active when the temperature varies between 18 and 29 °C. However, they are almost inactive below 10°C and above 30 °C. More than 1400 species of *Culicoides* can be identified around the globe. However, very few of them, less than 20, are capable of transmitting BTV to susceptible hosts [10]. The most potent vectors are *C. imicola*, *C. veripennis*, *C. fulvus*, *C. actoni*, *C. wadi*, *C. nubeculosis,* etc. So far, 29 BTV serotypes have been detected in the world [11]. The first 24 serotypes (serotypes 1–24) are categorized as typical, whereas the newer serotypes (serotypes 25–29) are categorized as atypical. Since 1998, the serotype distribution of BTV has been observed to be increasing, leading to critical outbreaks in Mediterranean countries. The same trend has been observed in northern and western Europe [12]. Even re-assortment leads to an antigenic shift in BTV, as observed in South Africa [13].

Most of the 23 serotypes in India are identifiable by serology or virus isolation [10,14]. Out of these identified serotypes, 15 serotypes (BTV-1 through 6, 9, 10, 12, 16 through 18, 21, 23 and 24) were detected using virus isolation, and 22 serotypes (BTV-1 through 20, 23 and 24) by serological assays [15].

Eastern and north-eastern India have not reported outbreaks or incidences of BT. However, the states cannot be termed as BTV-free, as a complex interaction always operates between the virus, vector, susceptible hosts and the environmental factors in the development of active bluetongue disease. Hence, the intrinsic interactions should be logically explored, with the aim of identifying the circulating virus and subclinical disease in the states. An earlier sero-epidemiological investigation was carried out in the eastern and north-eastern states of India [16]. Moreover, BTV isolation and characterization were carried out in sheep, goats and *Culicoides* sp. The sero-surveillance study was conducted from July 2001 until March 2017. We have now analyzed the sero-surveillance data using the Markov chain Monte Carlo (MCMC) method to calculate the positivity rates of various species across different states and agro-climatic zones. The objective of the work was to obtain updated and refined estimates of positivity rates that reflect the changing dynamics and varied prevalence of the disease across different regions. This will guide targeted interventions in bluetongue and support better decision-making in disease control and prevention.

## 2. Materials and Methods

### 2.1. Blood/Serum Samples

To prepare the serum samples, the collection of blood samples from sheep, goats and cattle (considered as susceptible animals) was carried out from different eastern and north-eastern (NE) states. As such, the samples belonged to states like Assam, Jharkhand, Meghalaya, Nagaland, Odisha, Sikkim, Tripura and West Bengal. The samples were collected from adult animals (more than one year in the case of cattle, and more than 3 months in the case of goats) of both sexes. Non-descript (indigenous) cattle, the Bengal breed of goats and non-descript (indigenous) sheep are prevalent in this region. Overall, the selection criteria for the animals were that they were apparently healthy individuals with no history of clinical bluetongue. The collected sera were stored at −20 °C. The details of the samples are narrated statewide as follows:

Assam: Serum samples (*n* = 729) were collected from sheep (*n* = 204), goats (*n* = 355) and cattle (*n* = 170) from different districts, viz., Golaghat, Karbi Anglong, Morigaon, Nalbari, Bongaigaon, Goalpara, Kamrup Metro and Kamrup Rural, covering various agro-climatic zones, viz., the Upper Brahmaputra Valley, Central Brahmaputra Valley, Lower Brahmaputra Valley, Hills, etc. The samples were collected from July to November and February to April of 2011–2012 and 2012–2013.

Jharkhand: A total of 480 serum samples were collected from apparently healthy sheep (*n* = 190), goats (*n* = 210) and cattle (*n* = 80) of different age groups from different districts of Jharkhand encompassing various agro-climatic zones, viz., the central and north-eastern plateau zone, the western plateau and the south-eastern plateau from July to November and February to April of 2013–2014.

Meghalaya: Sera were collected from apparently healthy sheep (*n* = 147), goats (*n* = 188) and cattle (*n* = 367) from different agro-climatic zones of Meghalaya covering various districts, viz., the East Khasi hills, the West Khasi hills, the South-West Khasi hills, the West Jaintia hills, Ri-Bhoi, the East Garo hills and the West Garo hills. The samples were collected during the post-monsoon months (July to November) of 2016. The details are given in Table 1.

Nagaland: A total of 50 serum samples were collected from apparently healthy free-range mithun (*Bos frontalis*) in their native tract in Nagaland, with abundant tropical rainforest, falling under three districts—Kohima, Phek and Tuensung—during the months of July and August 2013. Samples were collected from both calf (<2 yr old) and adult (>3 yr old) mithun of both sexes separately.

Odisha: A total of 364 serum samples were collected from apparently healthy sheep (*n* = 120), goats (*n* = 112) and cattle (*n* = 132) from different districts, viz., Cuttack, Mayurbhanj, Khurdah and Bhubaneswar, during the post-monsoon months (July to November) of 2013 and 2016.

Sikkim: A total of 330 serum samples were collected from apparently healthy ruminants, viz. sheep (*n* = 49), goats (*n* = 200) and cattle (*n* = 81) from different agro-climatic zones covering all the four districts of Sikkim, viz., East Sikkim, West Sikkim, North Sikkim and South Sikkim, during the months of June and July of 2017. The details of the sample collection are given in Table 2.

Tripura: The serum samples were collected from apparently healthy goats (*n* = 136) and cattle (*n* = 59) from West Tripura district during the months of July and August, 2016 and 2017.

West Bengal: A total of 1509 serum samples (sheep = 504, goats = 1005) were collected from different districts (agro-climatic zones) of West Bengal during the post-monsoon months (July to November), from 2010 through 2013, and subsequently tested.

### 2.2. Indirect Enzyme-Linked Immunosorbent Assay (i-ELISA) as a Tool of Sero-Surveillence

The collected serum samples were assessed using the indirect ELISA (i-ELISA) technique. The i-ELISA kit used in this study was an in-house-developed kit. This kit was developed and standardized by the Mukteswar center of the All India Network Programme on Bluetongue (AINP-BT) scheme of the Indian Council of Agricultural Research- Indian Veterinary Research Institute (ICAR-IVRI), Mukteswar Campus, Nainital (Uttarakhand), and generously gifted to our laboratory. However, the detailed procedure of the test has been described elsewhere [17,18]. In brief, the 96-well plates were coated by a recombinant BTV group (VP-7)-specific antigen to detect all the antibodies raised against all the serotypes. In the test, secondary immune conjugates (anti-sheep, anti-cattle and anti-goat) were used for detecting the anti-BTV antibodies in the samples.

### 2.3. Statistical Analysis

The Markov chain Monte Carlo (MCMC) method was used to calculate the positivity rates of various species across different states and agro-climatic zones.

## 3. Results

### 3.1. Assam

**Figure 1:**
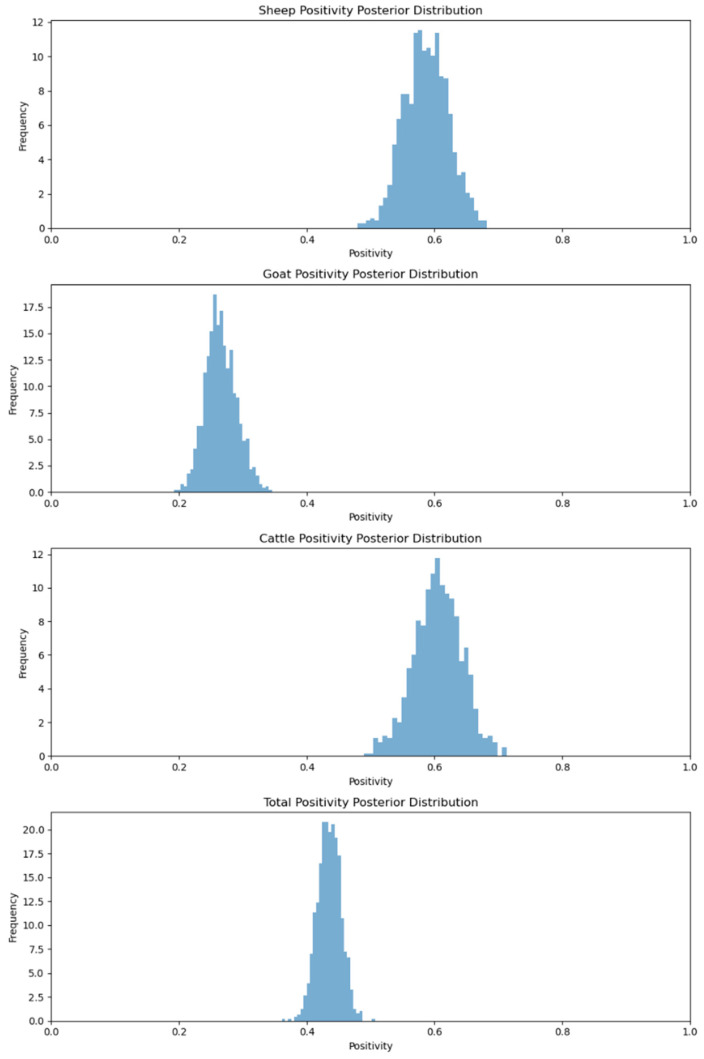
Animal species positivity posterior distribution for Assam.

The sero-monitoring serological testing of the Assam samples revealed different levels of anti-BTV antibodies in the sheep, cattle and goat sera. As such, 58.82% of the sheep, 31.79% of the goat and 70.00% of the cattle sera were positive for anti-BTV antibodies [18]. However, when the MCMC method was applied to calculating the positivity rates of various species, we noted that the provided data illustrate the seroprevalence of infections across different animal species—sheep, goats and cattle—based on the number of samples tested and the percentage of positive cases. The positivity rates vary significantly across species, with sheep showing a positivity of 58.82%, goats of 26.47% and cattle with the highest positivity of 60.58%. The overall positivity rate for all species combined is 43.48% (Figure 1). By using Bayesian inference, we can model the uncertainty in these positivity rates, allowing for the estimation of posterior distributions that account for both the observed data and prior beliefs. This approach helps to visualize and interpret the likely range of the true positivity rates within the population.

**Figure 2:**
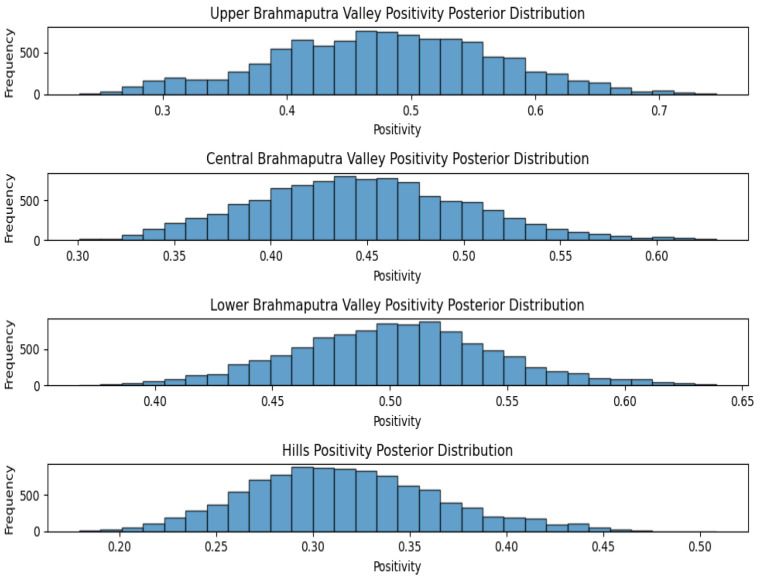
Agro-climatic zone positivity posterior distribution for Assam.

On the other hand, when the MCMC method was applied to the samples of various agro-climatic zones, Upper Brahmaputra Valley showed a positivity mean of 47.92%, whereas the Central Brahmaputra Valley positivity mean was 44.74%, the Lower Brahmaputra Valley positivity mean was 50.25% and the Hills positivity mean was 31.64% (Figure 2).

### 3.2. Jharkhand

**Figure 3:**
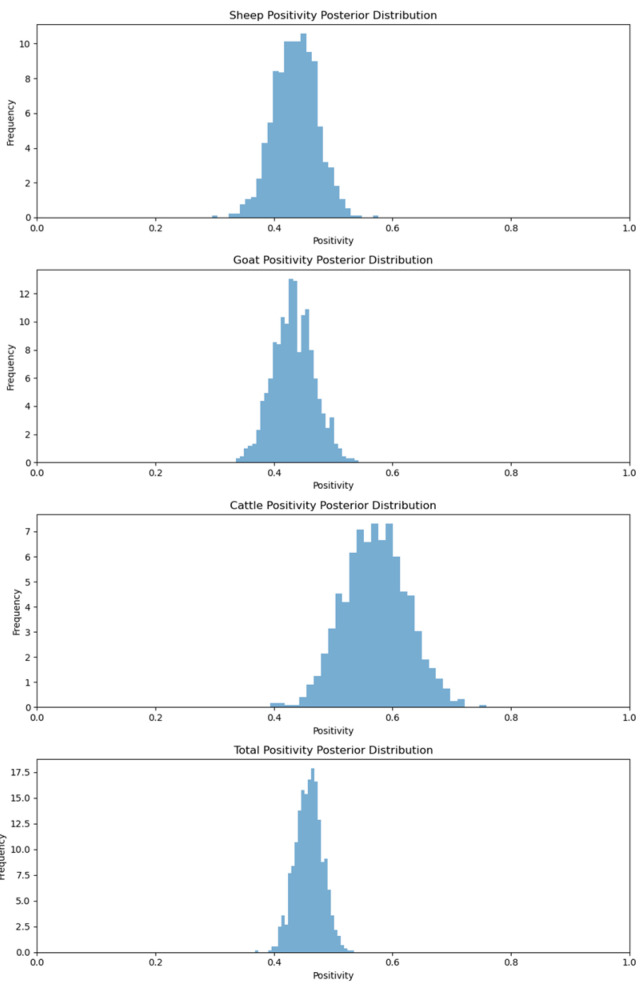
Animal species positivity posterior distribution for Jharkhand.

**Figure 4:**
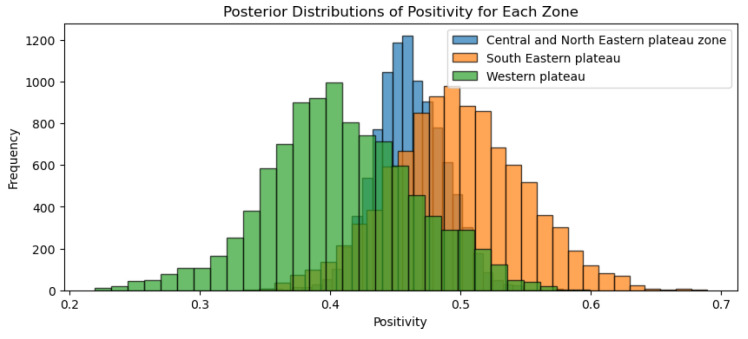
Agro-climatic zone positivity posterior distribution for Jharkhand.

The results of the sero-monitoring of the Jharkhand samples revealed that43.68% of sheep, 43.33% of goats and 57.50% of cattle possessed anti-BTV antibodies [19]. When the MCMC method was applied to calculating the positivity rates of various species, we found that the sheep positivity mean was 44%, while the goat positivity mean was 43% and the cattle positivity mean was 57% (Figure 3). On the other hand, when the MCMC method was applied to the various agro-climatic zones of Jharkhand, the results showed that the central and north-eastern plateau zone positivity mean was 45.79 percent, whereas the south-eastern plateau positivity mean was 50.73 percent and the western plateau positivity mean was 40.66 percent (Figure 4).

### 3.3. Meghalaya

**Figure 5:**
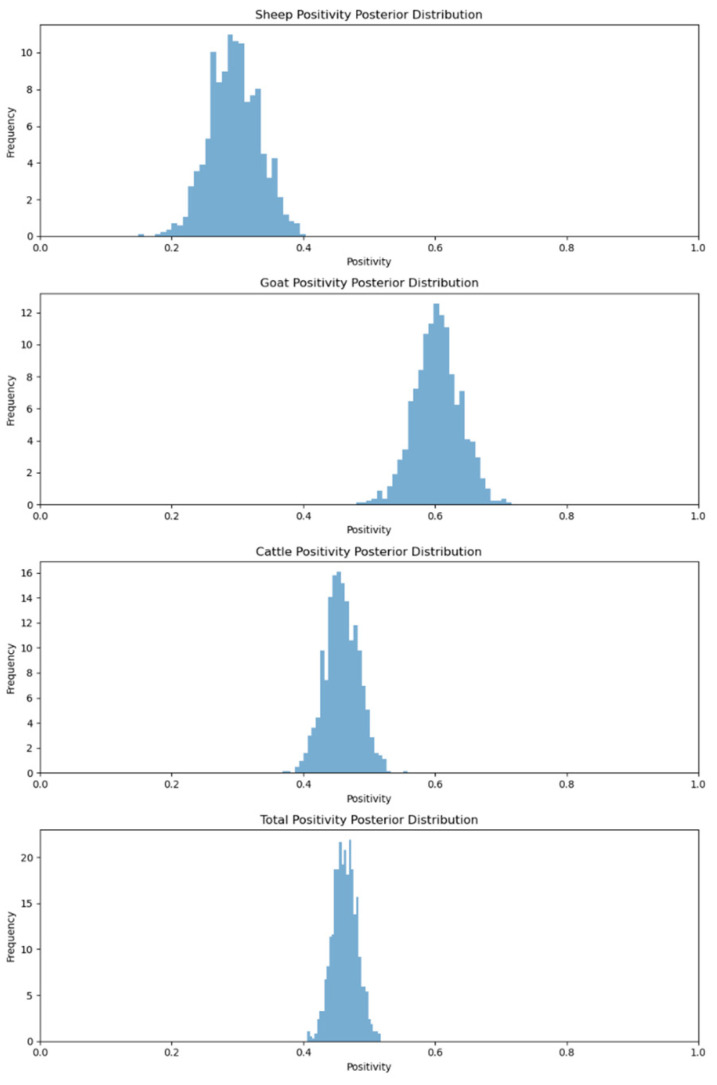
Animal species positivity posterior distribution for Meghalaya.

The Meghalaya samples revealed that 29.25% of sheep, 60.63% of goats and 45.77% of cattle had positivity for anti-BTV antibodies. Nongdharet al. [20] reported the highest prevalence of anti-bluetongue antibodies in the goat population when compared to cattle and sheep. Under the MCMC method, the seroprevalence data show distinct positivity rates across different animal species. Goats have the highest positivity rate at 60.00%, while sheep have the lowest at 29.00%. Cattle show a moderate rate of 45.00%. The overall positivity rate is 46.29% (Figure 5). Bayesian inference is used to model the uncertainty in these rates, providing a probabilistic view of the true infection levels. This approach helps in understanding the range of possible infection rates and assessing the spread of the disease within the tested population.

### 3.4. Nagaland

**Figure 6:**
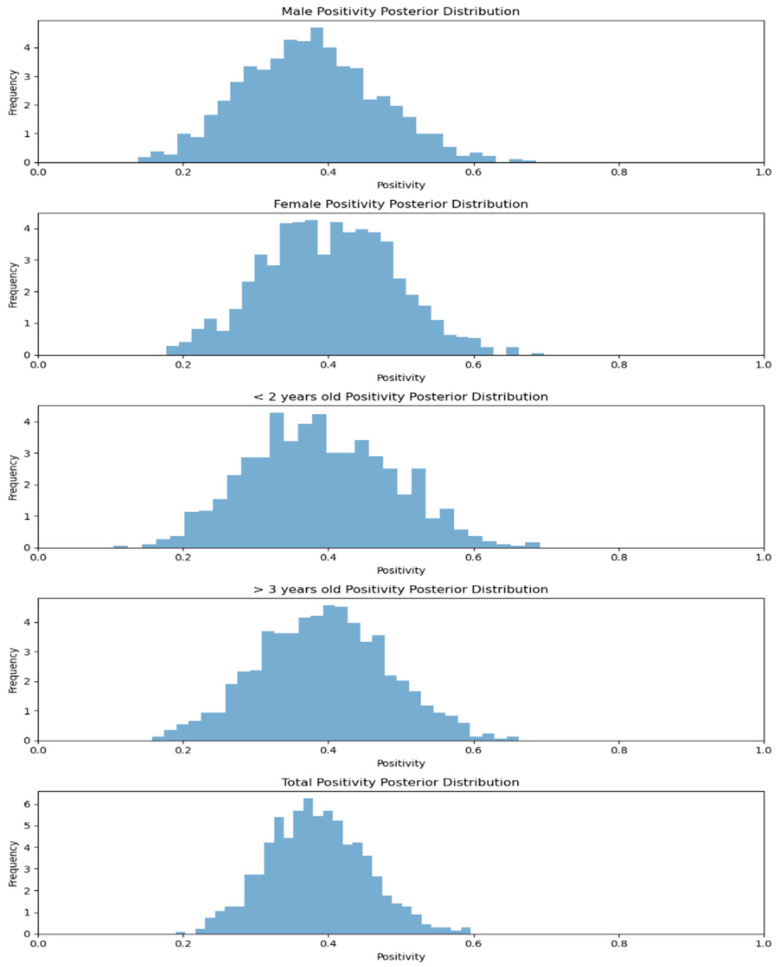
Sex-related and age-related positivity posterior distribution of mithun (*Bos frontalis*) for Nagaland.

The Nagaland mithun (*Bos frontalis*) samples (*n* = 50) revealed positivity of 36.36% and 39.26% in male and female animals, respectively. Furthermore, animals aged <2 yr had 37.50% positivity, but animals aged >2 yr showed 38.46% seropositivity [21]. When the MCMC method was applied to calculating the positivity rates of the two sexes and also of ages, males were at 36.36% and females at 39.28%, and animals younger than 2 years were at 37.50% and those older than 3 years at 38.46%. The overall positivity rate is 38.00%. Bayesian analysis of this data provides a probabilistic view of these rates, highlighting the uncertainty and range of possible true positivity levels for each category. This approach helps in understanding how infection rates vary with sex and age, offering insights into potential factors influencing disease spread (Figure 6).

### 3.5. Odisha

**Figure 7:**
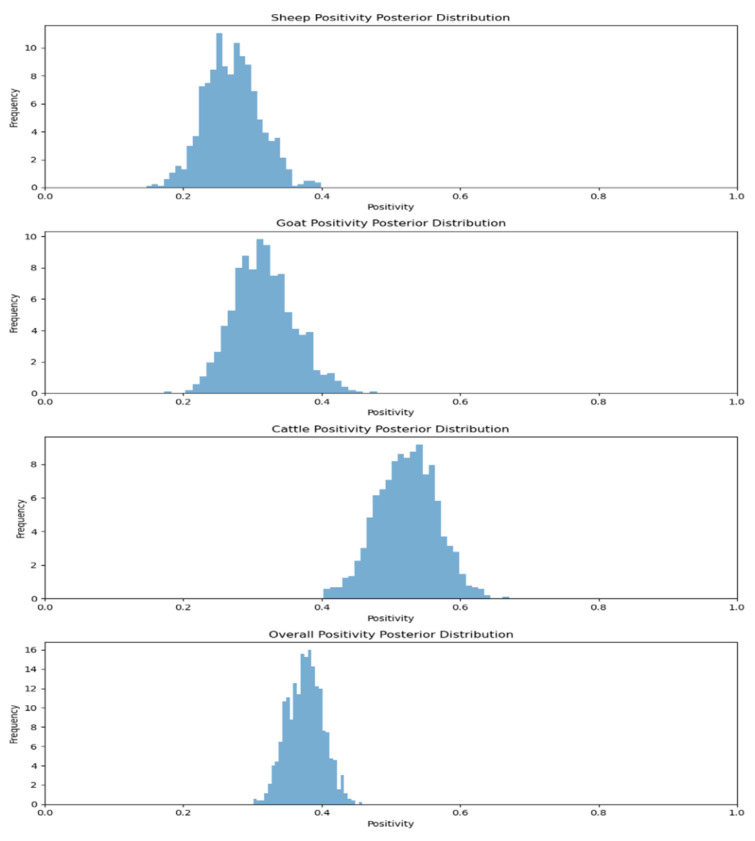
Animal species positivity posterior distribution for Odisha.

**Figure 8:**
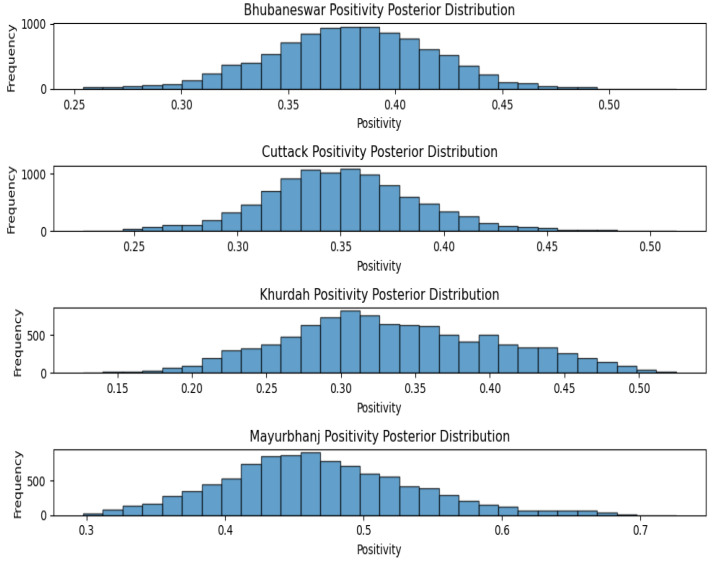
Agro-climatic zone positivity posterior distribution for Odisha.

Joardar et al. [22] reported the sero-surveillance data for the Odisha serum samples. Here, serum positivity was 26.66% for sheep, 31.25% for goats and 52.27% for cattle. When the MCMC method was applied to calculating the positivity rates of various species, the sheep positivity mean was 27.09 percent, the goat positivity mean was 31.28 percent and the cattle positivity mean was 52.79 percent (Figure 7). After applying the MCMC method to the different agro-climatic zones of Odisha, the Bhubaneswar positivity mean was37.91%, whereas the Cuttack positivity mean was 35.02%, the Khurdah positivity mean was 33.53% and the Mayurbhanj positivity mean was 46.77% (Figure 8).

### 3.6. Sikkim

**Figure 9:**
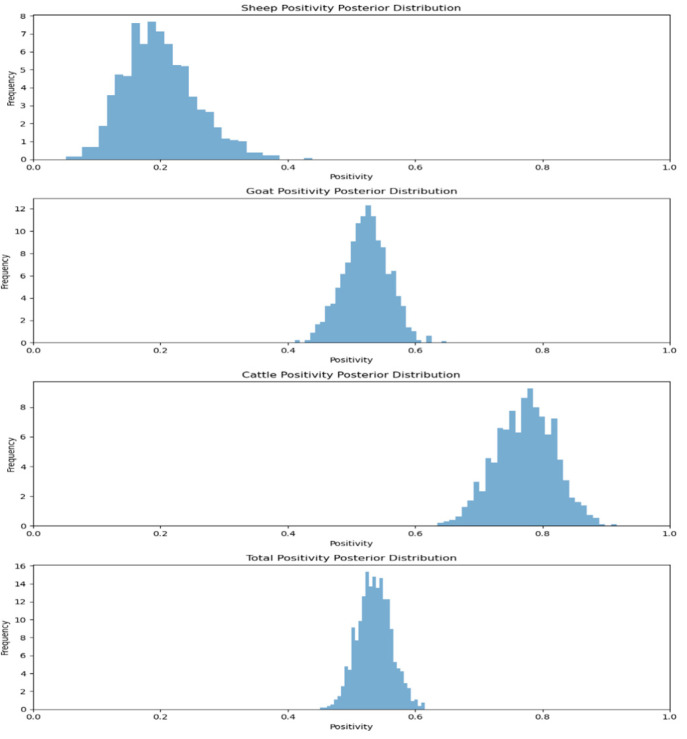
Animal species positivity posterior distribution for Sikkim.

A total of 330 serum samples from Sikkim were screened and reported seropositivity of 18.36%, 52.50% and 77.77% in sheep, goats and cattle, respectively [16]. When the MCMC method was applied to calculating the positivity rates of various species, it was found that the seroprevalence data for the anti-BTV antibodies across different species showed varying levels of positivity. Sheep exhibited a relatively low positivity rate of 18.36%, while goats had a much higher rate of 52.50%, and cattle had the highest rate at 77.77%. Overall, across all species, the positivity rate stands at 53.63% (Figure 9). By applying Bayesian inference, the uncertainty in these positivity rates is modeled, providing a clearer understanding of the true infection prevalence in the population. This approach helps in visualizing the potential spread of the disease among the tested animals.

**Figure 10:**
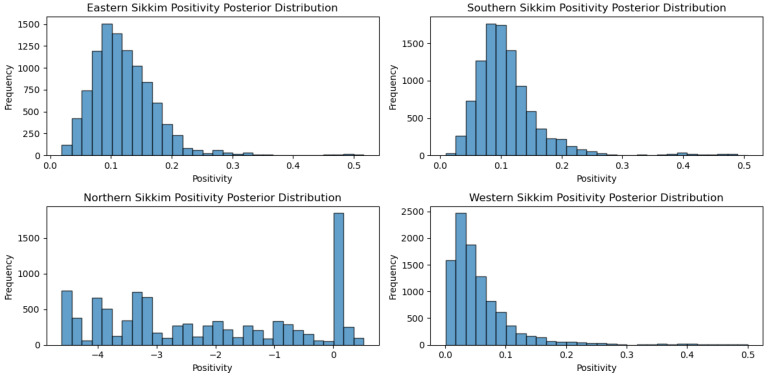
Agro-climatic zone positivity posterior distribution for Sikkim.

On the other hand, when the MCMC method was applied to the different agro-climatic zones of Sikkim, the eastern Sikkim positivity mean was12.12 percent, the western Sikkim positivity mean was 5.86 percent, the southern Sikkim positivity mean was 11.12 percent and the northern Sikkim positivity meanwas-217.83 percent (Figure 10).

### 3.7. Tripura

**Figure 11:**
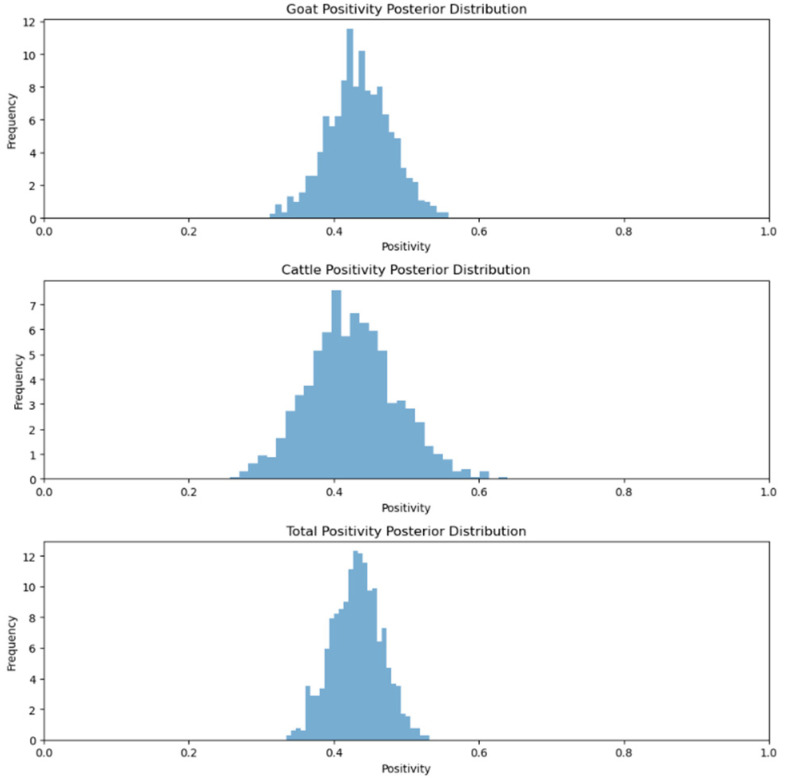
Animal species positivity posterior distribution for Tripura.

In the sero-monitoring of the Tripura samples, 43.88% of goat samples and 42.37% of cattle samples were found to be positive for anti-BTV antibodies [23]. When the MCMC method was applied to calculating the positivity rates of various species, the seroprevalence data for the anti-BTV antibodies in the goats and cattle indicated positivity rates of 43.38% for goats and 42.37% for cattle, with an overall positivity rate of 43.07% across all samples (Figure 11). Bayesian inference is used to model the uncertainty in these rates, helping to better estimate the true infection prevalence within the population. This approach provides a probabilistic view of the positivity rates, offering insights into the potential variability and spread of the disease among the animals tested.

### 3.8. West Bengal

From the data collected between July and December of 2010 through 2013 regarding the serum samples (*n* = 1509) from the different agro-climatic zones of West Bengal [24], we can observe several key trends:

1. New Alluvial Zone:
  - A total of 122 sheep and 332 goat samples were collected.
  - Out of these, 48 samples tested positive (39.34% positivity) in sheep.
  - In goats, 137 samples tested positive, with a 41.26% positivity rate.
2. Red Laterite Zone:
  - A higher number of sheep samples (250) were collected compared to goats (120).
  - For sheep, 79 samples were positive, yielding a 31.60% positivity rate.
  - For goats, 120 positive samples were detected from 553 tested, giving a relatively lower positivity rate of 21.69%.
3. Coastal Saline Zone:
  - A total of 132 sheep and 120 goat samples were collected.
  - Sheep showed a positivity rate of 30.30%, while goats had a higher rate of 37.16%.
4. Overall Summary:
  - Across all zones, a total of 504 samples were collected, with 167 positive cases and an overall positivity rate of 33.13% for sheep.
  - In the case of goats, 1005 samples were collected, with 304 positive cases and a lower overall positivity rate of 30.24%.

### 3.9. Observations

**Figure 12:**
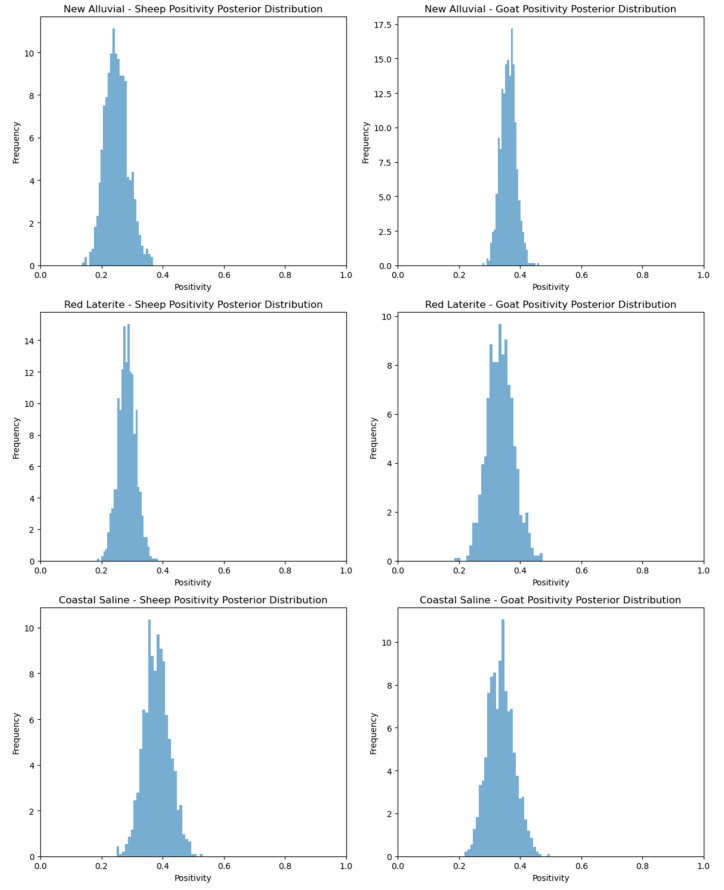
Agro-climatic zone-related positivity posterior distribution for West Bengal, July 2010–December 2013.

- Positivity rates are generally higher in the New Alluvial Zone than in the other zones for both sheep and goats.
- The Red Laterite Zone showed a notably lower positivity rate, especially for goats, at 21.69% (Figure 12).
- The Coastal Saline Zone has comparable positivity rates for sheep and goats, with goats showing a slightly higher rate.
- Overall, goats tend to have a slightly higher positivity rate for bluetongue antibodies across zones than sheep.

These data highlight the potential regional variations in bluetongue virus (BTV) exposure and immune response in livestock, emphasizing the need for targeted control measures based on agro-climatic zones.

## 4. Comparison of Species Positivity Rates Across States

**Figure 13:**
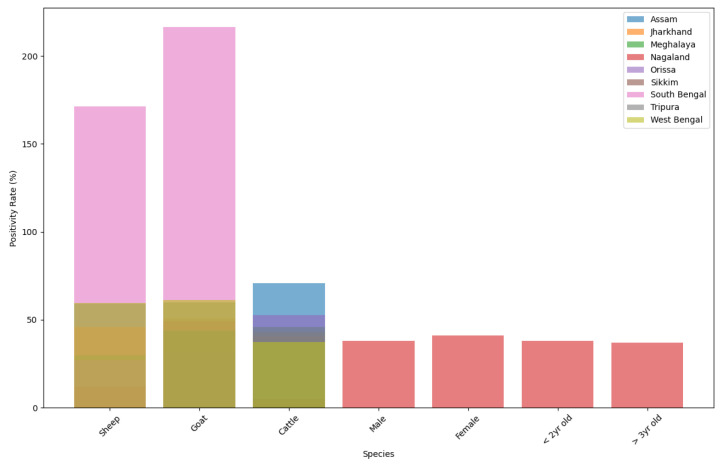
Comparison of species positivity rates across states.

**Figure 14:**
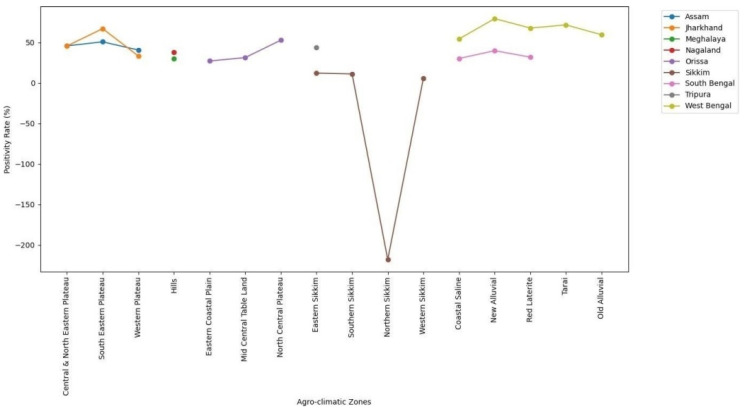
Comparison of positivity rates across states and agro-climatic zones.

From the comparison graphs of species (Figure 13) and agro-climatic zones across different states (Figure 14), we observed the following:

### 4.1. Species Positivity Rates

Sheep: The positivity rates varied across states, with some states showing higher rates (e.g., South Bengal) compared to others (e.g., Sikkim).

Goats: Similarly, the positivity rates for goats varied across states, with notable differences between states like South Bengal and Sikkim.

Cattle: The positivity rates for cattle exhibited variations across states, indicating differences in disease prevalence and management practices.

### 4.2. Agro-Climatic Zone Positivity Rates

Different states, different zones: The positivity rates across different agro-climatic zones varied significantly, indicating geographical variations in disease prevalence and environmental factors influencing disease transmission.

Statewide variation: Each state exhibited unique patterns in positivity rates across agro-climatic zones, reflecting regional differences in disease dynamics.

## 5. Discussion

This sero-surveillance study conducted on the susceptible ruminants of the eastern and north-eastern states of India showed the presence of substantial amounts (40% on average) of anti-BT antibodies in the sera, indicating a prevalence of circulating BTV in the region [16]. This corroborates the findings of earlier studies that reported 47% overall seropositivity for the ruminants of Maharashtra [25]. Comparatively high seropositivity was observed in goats (76%) and cattle (60.5%) in Haryana, Himachal Pradesh and Rajasthan [26] and cattle (70%) in Punjab [27]. However, other studies found low positivity rates (23.4%) in cattle sera in Haryana, Punjab, Himachal Pradesh and Rajasthan [28] and goat sera (5.1%) in Kerala [29].

We have analyzed the sero-surveillance data using the Markov chain Monte Carlo (MCMC) method to calculate the positivity rates of various species across different states and agro-climatic zones. In our study on bluetongue virus (BTV) seroprevalence, the use of posterior positivity distribution is actually crucial for several reasons, viz., accurate seroprevalence estimation, species-specific insights, regional analysis, enhanced decision-making and epidemiological insights. The posterior positivity distribution helps in accurately estimating the seroprevalence of BTV among different species and regions. For example, in Meghalaya, the adjusted seroprevalence rates provide a clearer understanding of the infection spread among goats and cattle. By refining raw positivity rates, the method ensures that our estimates consider various uncertainties and sampling errors, leading to more reliable results. Our study reveals species-specific insights, such as the calculated positivity means for goats and cattle in Tripura (43.67% and 43.15%, respectively). These insights are essential for understanding which species are more affected and for guiding targeted interventions. In Nagaland, sex-wise and age-wise posterior positivity distributions for mithun highlight differences, although not statistically significant, providing a comprehensive view of infection dynamics. The method allows for detailed regional analysis, as seen in Sikkim, where significant variations in seroprevalence across different agro-climatic zones were observed. Such insights are vital for developing region-specific strategies for disease control. In Jharkhand, the posterior positivity distribution across species and agro-climatic zones highlights regional differences, informing local health interventions and resource allocation. The comprehensive understanding provided by the posterior positivity distribution supports better decision-making in disease control and prevention. By knowing the precise distribution of positivity, authorities can prioritize resources and interventions more effectively. This method enhances our ability to make informed decisions regarding vaccination programs, surveillance and other control measures. Detailed posterior distributions enable us to track the temporal and spatial dynamics of BTV outbreaks. This epidemiological insight is crucial for understanding the factors driving the spread of the disease and for predicting future outbreaks. By analyzing posterior positivity distributions, we can identify patterns and trends in BTV seroprevalence, guiding future research and public health strategies.

It is to be mentioned here that BTV was isolated from sheep [30], goats [31] and *Culicoides* midges [32] of West Bengal state. Hence, it may be anticipated that in this region, BTV was in circulation in animals and vectors in eastern India as well as in north-eastern states. Although BT is considered an active infection, sheep, goats and cattle may act as reservoirs of BTV as detected in other parts of the world [33].

In this background, from the disease prevalence based on statistical analysis through MCMC, updated and refined estimates of positivity rates have been obtained that reflect the changing dynamics and varied prevalence of the disease across different regions. To our knowledge, this is the first time that the MCMC method has been successfully used to analyze posterior positivity rates in BTV seroprevalence. Since no comparable data (based on MCMC) are available for BT, we failed to incorporate any related works in the Section 5. The use of posterior positivity distribution has provided significant insights into the massive seroprevalence of BTV in the animals of the eastern and north-eastern states of India, constituting a wakeup call for outbreak preparedness. However, our study has limitations and biases in terms of the uneven sample collection from different states and animal species. As such, we expect better results (outcomes of an MCMC analysis) in similar studies that include an even and large number of samples in the future.

BT outbreak preparedness is quite relevant (also pertinent) in the context of recent occurrence of BTV in its rejuvenate forms in different parts of the world, including India [34]. It may be mentioned here that recent surveillance studies proposed some atypical/novel serotypes of BT in different countries [35]. Currently, northern Europe is facing the menace of newer strains of BTV, threatening sheep and cattle husbandry [36]. In the last year, BTV-3 was reported in the Netherlands, which subsequently spread to Belgium, Germany and south-east England. Interestingly, in the last year, a new strain of BTV-8 has again been detected in France, with widespread distribution. A wind-borne incursion of infected *Culicoides* into Great Britain from northern Europe has even been anticipated [37].

## 6. Conclusions

Some of the salient features and outcomes of the statistical analysis in the present investigation may be highlighted as follows:

(i) In Meghalaya, the adjusted seroprevalence rates provided a clearer understanding of the infection spread among goats and cattle. By refining raw positivity rates, the method ensures that our estimates consider various uncertainties and sampling errors, leading to more reliable results. The species-specific posterior positivity distributions showed nearly equal infection rates among goats and cattle, aiding in the development of region-specific interventions. The sex-wise and age-wise posterior positivity distributions for mithun revealed differences in BTV seroprevalence, although not statistically significant.
(ii) This comprehensive view helps in understanding the infection dynamics and supports targeted health interventions in Nagaland.
(iii) In Jharkhand, the posterior positivity distribution across species and agro-climatic zones highlighted regional differences, providing insights into local disease dynamics.
(iv) In Tripura, the species-specific posterior positivity distributions showed nearly equal infection rates among goats and cattle, aiding in the development of region-specific interventions.

In the present study, an MCMC analysis enhanced our understanding of infection dynamics, species-specific insights and regional analysis by accurate seroprevalence estimation. Therefore, as a final comment, we would like to add here that as a powerful computational tool, MCMC could be used for enhanced decision-making and epidemiological insights in addition to control, prevention and disease preparedness for bluetongue in the future. Previously hidden in normal analysis, these insights could be explicitly expressed via statistical analysis, especially using the MCMC technique, which is a powerful computational tool in modern-day research.

## Figures and Tables

**Table 1:** Details of the collected samples covering different agro-climatic zones of Meghalaya.

Sl. No.	District	Agro-Climatic Zones	Collection Area	Sample Number
1	Part of East Khasi Hills	Humid with moderately warm summers and severe, cold winters, featuring high rainfall (2800–6000 mm)	Mawngap-Rim, Mawmyrsiang, Rangskhen	431
2	West Khasi Hills & East Garo Hills	Humid & moderately cold in winter, with high rainfall (2800–4000 mm)	Mawthadraishan, Mawkohngei, Nongshillong, Bolkinggre, Ampangdanggre	121
3	Ri-Bhoi, West Jaintia Hills, Part of East Khasi Hills & West Garo Hills	Warm & humid with medium rainfall (1270–2032 mm)	Patharkhmah, Kyrdemkulai, Sonidan, Saitsama, Laitkseh, Mawkynthih, Rongram, Asanang	140
4	South-West Khasi Hills	Humid & hot with high rainfall (2800–4000 mm)	Nonglang, Mawlangwir	10
		Total		702

**Table 2:** Details of serum samples collected from different districts of Sikkim.

Sl. No	Name of District	Agro-Climatic Zones	Sheep	Goat	Cattle	Total
1	East Sikkim	Moderately cool & cool insome parts	-	37	-	37
2	West Sikkim	Warm & moderately cool insome parts	38	-	-	38
3	North Sikkim	Cool & moderately cool	-	19	-	19
4	South Sikkim	Warm & moderately cool	11	144	81	236
		Total	49	200	81	330

## Data Availability

All the data and materials used to reach the conclusion of this study are available from the corresponding author on request.
